# Acetazolamide and Thyroid-Associated Ophthalmopathy; a Preliminary Tested Hypothesis in a TertiaryReferral Center

**Published:** 2013

**Authors:** Gholamreza Khataminia, Farshad Ostadian, Mohammad Noroozzadeh, Mahmoud Latifi, Masoud Khataminia

**Affiliations:** 1 Department of Ophthalmology, Ophthalmology Research Center, Jundishapur University of Medical Sciences, Ahvaz, Iran; 2 Department of Health, Diabetes Research Center, Jundishapur University of Medical Sciences, Ahvaz, Iran

**Keywords:** Thyroid-associated ophthalmopathy (TAO), Acetazolamide, VISA score, Orbital pain, Eyelid edema, chemosis, Injection of the eyelids, Conjunctival injection

## Abstract

This study evaluated the effect of acetazolamide on thyroid-associated ophthalmopathy (TAO). Patients with a VISA classification index equal to or more than four were enrolled in the study and were randomly assigned into two groups. In both groups, treatment was initiated using prednisolone. Patients in the case group received acetazolamide tablets 250 mg daily in addition to prednisolone. Three months later, the VISA inflammatory score of patients in both groups were determined. Subsequent to intervention with acetazolamide, the VISA inflammatory score of patients in the case group were reduced as follows; orbital pain (57.1% versus 41.7%, P=0.736), eyelid edema (42.8% versus 27.1%, P=0.67), chemosis (53.3% versus 33%, P=0.31), injection of the eyelids (60% versus 41.6%, P=0.342), and conjunctival injection (50% versus 46.13%, P=0.73). However, these reductions were not statistically significant when compared with those observed in the control group (P=0.246). In conclusion, the effect of acetazolamide on all the parameters of the VISA inflammatory score was examined independently. All patients in the case group revealed a reduction in VISA inflammatory score following intervention. However, these reductions were not statistically significant. Further studies with large sample sizes are required.

## INTRODUCTION

Thyroid-associatedophthalmopathy (TAO) should be considered as an autoimmune inflammatory disorder, which is associated with Graves’ hyperthyroidism, Hashimoto’s thyroiditis, primary hypothyroidism, and euthyroidism with rates of approximately 90%, 3%, 1%, and 6%, respectively (1). Most patients with TAO reveal minimum symptoms, and only almost 4% determine significant clinical manifestations (2). Orbital fibroblasts play an essential role in the inflammatory process of TAO. Formation of specific surface receptors and expression of proinflammatory genes play an important role in this inflammatory process. During the inflammatory process of TAO, production of interleukin 6 and 8 and prostaglandin E2 increases the synthesis of hyaluronan glycosaminoglycan by approximately one hundred fold. This causes orbital fibroblasts and fat cells to change the connective tissue structure of the orbit (3). Ultimately, increased glycosaminoglycan accumulation sets off an increase of fluid retention in the orbital space (4), which increases the thickness of extraocular muscles, fat, retrobulbar, and connective tissue and results in fibrotic changes in the muscle (1-4).

 On the other hand, TAO is classified according to activity or severity. Differentiation between severity and activity is imperative because each requires different treatments. Werner and NOSPECS classifications are based on disease severity, while Mouritis and VISA inflammatory score classifications are based on disease activity (5). According to the VISA classification, scoring of the findings are as follows (6,7):

a. Orbital pain: painless orbit, 0; pain at rest, 2; pain when moving eyes, 1.

b. Chemosis: chemosis that spreads beyond the gray line, 2; chemosis that does not extend beyond the gray line, 1; absence of chemosis, 0.

c. Eyelid edema: eyelid edema that does not change the superficial anatomy of eyelid and does not cause drooping or hanging disruption, 1; eyelid edema that results in changes in anatomy and hanging of the eyelid, 2.

d. Conjunctivalinjection: presence of conjunctival injection 1; absence of conjunctival congestion, 0.

e. Eyelid injection: presence of eyelid injection, 1; absence of eyelid injection, 0.

 Most patients with mild TAO only requireconservative treatment. In patients with moderate to severe active orbitopathyor optic nerve involvement, oral corticosteroids such as prednisolone 1mg/kg are recommended for 2-4 weeks until an appropriate response occurs, after which they are gradually tapered off the medication. If the VISA inflammatory score is less than 4, only conservative management is offered; however, if the score is more than four, treatment with prednisolone is recommended (7). If glycosaminoglycan accumulation occurs resulting in fluid retention, diuretics such as carbonic anhydrase inhibitors may prove beneficial for volume reduction in addition to corticosteroid compounds, which decrease inflammation (8). Since compounds such as acetazolamide are easily absorbed through the gastrointestinal tract, their maximum effects are achieved within 2 hours following consumption and persist for 12 hours following administration. Because these compounds are excreted through the kidneys and tubular secretion, their use should be restricted in patients with renal failure (9). As a matter of fact, there is a limited study on the effectiveness of acetazolamide on TAO; therefore, we planned to test this hypothesis in a tertiary referral center at Imam Khomeini Hospital, Jundishapur University of Medical Sciences (JUMS), Ahvaz, Iran.

## METHODS

The inclusion criteria for this study were VISA inflammatory score more than 4, and the exclusion criteria were contraindication of acetazolamide or VISA inflammatory score less than 4. Informed consent was obtained and the protocol has been approved by the ethical committee of JUMS. 

 Both cases and controls were randomly assigned based on VISA inflammatory score.Twenty patients were each enrolled in the case group and in the control group, respectively. Patients were examined for eyelid edema, conjunctival and eyelid injection, proptosis, and visual acuity. Fundoscopy and intraocular pressure were checked in all patients. Patients with VISA inflammatory score more than 4 were randomly divided into two groups. Prednisolone was initiated in both groups, and acetazolamide tablets 250 mg daily in four divided doses were initiated in the case group. After three months, the of patients in both the groups were re-evaluated, and the scoring was performedaccordingly.

## RESULTS

The mean age of patients in the control group was 38.2±9.8 years while in case group was 42±10.2 years (P=0.212). In the control group, 6 (30%) patients were men and 14 (70%) were women; while in the case group, 8 (40%) patients were men and 12 (60%) were women. 

 The effect of oral acetazolamide in the case group was evaluated in terms of changes in the VISA inflammatory score. In the control group, the scoring criterion prior to treatment with prednisolone was 5.16±0.65 andfollowing three months of treatment with prednisolone was 4.5±0.97. The VISA scoring system was used to determine the role of prednisolone in reducing inflammatory markers prior and after treatment. The results revealed a significant association between use of corticosteroids and inflammatory changes in the VISA scoring system (P=0.008). In the case group, the scoring average based on inflammatory indices before and after the intervention were 5.05±0.55 and 4.05±0.96, respectively. Although the use of acetazolamide decreased the VISA inflammatory score of patients in the case group, no significant difference was observed between the VISA inflammatory score of patients in the two groups (P=0. 246).

 In our study, we examined the effects of acetazolamide on each of the five inflammatory indices of the VISA scoring system. Three patients in the control group and six patients in the case group revealed improvement in eyelid edema. Although the rate of eyelid edema improvement in the case group was greater than that in the control group, the difference was not statistically significant (42.8% versus 27.1%, P=0.677).

 Orbital pain improvement was reported in five (41.7%) patients in the control group and eight (57%. 1) patients in the case group. Although the degree of orbital pain improvement in the case group was greater than that in the control group, the difference was not statistically significant (P=0.736).

 Conjunctivalchemosis was improved in four (33%) patients in the control group and eight (53.3%) patients in the case group. Even though the degree of improvement in conjunctivalchemosis in the case group was greater than that in the control group, the difference was not statistically significant (P=0.311).

 Eyelidinjection was improved in five (41.6%) patients in the control group and nine (60%) patients in the case group. However, the degree of improvement was not significantly different between the two groups (P=0. 342). Conjunctival injection was improved in six (46.1%) patients in the control group and seven patients in the case group (50%); however, the degree of improvement was not significantly different between the two groups (P=0. 736).

## DISCUSSION

With regard to the pathogenesis of TAO due to fluid retention of hydrophilic materials such as hyaluronan glycosaminoglycan in the orbital space (6-9), in the study, acetazolamide had been administrated as a diuretic to reduce interstitial fluid. This study was designed to assess the hypothesis regarding effectiveness of acetazolamide on the active phase indices of TAO since limited research was found in this regard. 

 Although there are few reports revealed that acetazolamide may exacerbate thyrotoxicperiodic paralysis bouts (10,11), we did not record any acetazolamide related side effects in our follow up. However it is pertinent to follow the cases in a longer duration.

In our study, the effect of acetazolamide on all the parameters of the VISA inflammatory score was examined. Recovery and decrease in the VISA inflammatory score following the intervention was noted in most subjects; however, no significant differences were observed between the two groups with respect to the reduction in VISA inflammatory score. Although the results obtained from the control group in the context of the effectiveness of prednisone in the inflammatory phase of TAO reduced the VISA inflammatory score, inflammatory indices were statistically significant (P=0.008). When comparing the case and control groups in terms of acetazolamide intervention during the three-month period revealed no significant differences (P=0.246), the reduction in the VISA inflammatory score was greater in the case group. 

 Our limitations included low incidence of TAO and small sample size, therefore similar studies with a larger sample size, longer duration of the active phase of TAO, and non-inflammatory side effects are necessary in order to conclude the effects of acetazolamide on thyroid-associated ophthalmopathy.
